# Acceptance and impact of Technology-Enabled Care (TEC) among community-dwelling older adults: A rapid review

**DOI:** 10.1371/journal.pdig.0001625

**Published:** 2026-08-04

**Authors:** Abraham Sahilemichael Kebede, Ann-Marie Morrissey, Kevin Moore, Katie Crowley

**Affiliations:** 1 Lero, the Research Ireland Centre for Software, University of Limerick, Limerick, Ireland; 2 Ageing Research Centre, Health Research Institute, University of Limerick, Limerick, Ireland; 3 School of Allied Health, University of Limerick, Limerick, Ireland; 4 Ei Electronics, Shannon, Ireland; University of Pittsburgh School of Medicine, UNITED STATES OF AMERICA

## Abstract

Technology-Enabled Care (TEC) represents a broad approach to supporting older adults’ quality of life, ageing in place, and independent living using digital, assistive, monitoring and communication technologies. This review aimed to synthesise existing evidence on factors influencing TEC acceptability and impact among community-dwelling older adults. We employed a rapid review methodology guided by the PCC framework (Population: community-dwelling older adults 55 years and above and carers; Concept: TEC acceptability and impact; Context: community or home). Three databases (PubMed, CINAHL, and Web of Science) were searched, yielding 7,550 records, of which 41 primary studies were included. Most studies were conducted in Europe (49%), used qualitative methods (51%), and focussed on surveillance and safety technologies (*n* = 16). Factors influencing TEC acceptability were synthesised into five domains: (1) individual characteristics and readiness, (2) perceived values and trade-offs, (3) sociocultural and relational dynamics, (4) contextual and structural enablers, and (5) technology design and appropriateness. A cross-cutting interpretive analysis identified four mediating mechanisms shaping TEC acceptance: trust, the tension between intrusiveness and autonomy, stigma and identity, and perceived control. Reported impacts of TEC included enhanced safety and independence, improved communication and social connection, health promotion and prevention of adverse events, and increased efficiency and cost-effectiveness of care. Overall, TEC acceptability is shaped by dynamic interactions between individual, social and contextual factors across the life course. Maximising its impact requires a human-centred, participatory approach that recognises heterogeneity among older adults and actively involves users and caregivers throughout the design and implementation process.

**OSF registration:** https://doi.org/10.17605/OSF.IO/2ENZ7.

## Author summary

As the global population ages, many older adults wish to remain living safely and independently in their own homes for as long as possible. Technology-Enabled Care (TEC), such as smart home systems, wearable devices, and remote monitoring tools, offers new ways to support daily living, improve safety, and reduce pressure on health and social care systems. However, not all older adults accept or benefit equally from these technologies. We carried out a rapid review of 41 studies published between 2015 and 2025 to better understand what helps or hinders older adults in using TEC, and what impact it has on their lives. We found that acceptance of these technologies depends on many factors, including digital skills, personal health, perceived benefits, privacy concerns, cultural attitudes, costs, and whether the design is simple and comfortable. Underlying issues such as trust in technology, the balance between independence and surveillance, stigma, and a sense of control also play key roles. The review also showed that TEC can enhance safety, independence, communication, and health management, and may reduce healthcare costs. At the same time, challenges such as privacy worries, technical failures, or feelings of dependency discourage use. Our findings highlight that successful adoption of TEC requires a human-centred and participatory approach. Older adults and caregivers must be involved throughout the design and implementation process to ensure technologies are trustworthy, accessible, and truly supportive of ageing in place.

## Introduction

Population ageing is a global phenomenon [1]. Both the absolute and relative population proportions are projected to increase, from 1.1 to 1.4 billion by 2030, and from 12% in 2015 to 22% in 2050, respectively [2]. Common functional, health, and societal challenges, including multimorbidity, chronic diseases, mobility limitations, and social isolations are related to such demographic shift, increasing the demand for long-term and home-based care, further stretching the existing health and social care services (e.g., workforce, economic needs, etc) [3,4]. As a result, there is increasing emphasis on developing innovative, scalable, and sustainable models of care that support older adults to live independently while maintaining quality of life.

Ageing in place has become a key policy and health and social care priority, reflecting the strong preference of older adults (over 80%) to remain living at home and in the community for as long as possible, with substantial evidence showing associated benefits for independence, social connection, reduced social isolation and depression, cognitive functioning and overall quality of life [5–9]. These benefits are reinforced through home environment adaptations via a network of supportive services, community ties, and social connections within familiar surroundings. Conversely, the support needs of older adults vary considerably across individuals and over time, depending on their ability to perform Activities of Daily Living (ADLs) and Instrumental ADLs. Living arrangements (e.g., living alone versus with others), health status and multimorbidity, functional trajectories across the life course, availability of informal care, socioeconomic resources, digital literacy, and housing and infrastructural contexts are all key factors [10,11].

Technology-enabled care (TEC) is a convergence of digital health, digital media and mobile telecommunication technologies to provide convenient, accessible and cost-effective care and support, and encompasses various technologies, including telemedicine, telehealth, telecare, remote monitoring, digital health and mHealth [12,13]. It has therefore been proposed as a vital mechanism to support ageing in place by addressing heterogeneous and changing care needs, improving access to appropriate forms and intensities of care, reducing care burden and associated costs, improving social and physical capabilities of older adults, and enhancing independence and safety [14–16]. TEC contributes to more sustainable models of care by enabling earlier identification of risk, remote support, timely intervention, and more efficient allocation of care resources. These potential contributions depend on technical and organisational capabilities such as data sharing, interoperability, and integration with existing care pathways, and the capacity to identify changing care needs over time. In light of this potential, both adoption and the need for TEC have significantly increased over the past years. For example, a 2020 survey in the US reported how the adoption and use of telehealth jumped from 4.6% pre-pandemic to 21.1% [17].

However, the availability and expansion of TEC do not necessarily translate into meaningful uptake, ongoing engagement, or successful implementation in everyday care. Older adults may experience barriers related to digital literacy, confidence, perceived usefulness, affordability, privacy, trust, usability, physical or cognitive limitations, and concerns that technology may replace rather than supplement human support. Technologies designed to support safety, independence, or ageing in place may also not be used as intended if they do not align with older adults’ preferences, routines, care relationships, home environments, or wider service contexts. Understanding acceptance, adoption, and sustained use is therefore essential for explaining why TEC interventions may succeed, fail, or produce variable impacts in practice.

Despite growing evidence on the potential benefits of TEC, its real-world impact, depends on a complex behavioural trajectory that is often oversimplified in the literature. Acceptance, adoption, and sustained use are sometimes treated as interchangeable outcomes, although they represent distinct stage of engagement [18]. Willingness to use a technology does not necessarily lead to actual uptake and initial adoption does not guarantee long-term integration into everyday routines. Acceptance represents a pre-implementation psychological state, reflecting an individual’s willingness or intention to use a technology based on perceived usefulness and ease of use, but does not in itself guarantee actual utilisation [19]. Adoption and sustained use (post-adoption) denote meaningful interaction with a technology, whereby intention translates to observable use or long-term integration into everyday practices beyond initial experimentation or novelty [20,21]. Importantly, these outcomes are not linear but are shaped by interrelated factors operating across individual, social, and environmental levels. In this review, acceptance is examined as the primary behavioural foundation, while adoption and sustained use are treated as distinct constructs that contextualise the longer-term viability of TEC interventions.

Building on this distinction, evidence from previous reviews indicates that the success of TEC depends less on technical sophistication and more on alignment with users’ capabilities, motivations, and everyday contexts [22]. Despite digital health interventions impact, acceptance and use are constrained by low digital literacy, limited self-efficacy, concerns around trust and privacy, and poor integration with existing services. Importantly, the relationship between acceptance and impact is mediated by the type of technology employed: for eHealth and mHealth platforms, impact is shaped by digital self-efficacy and cognitive load [23,24]; for wearable devices, by social and identity-related factors such as stigma and personal relevance [25]; and for assistive and monitoring technologies, particularly among individuals with cognitive impairment, by the presence of ‘*invisible*’ usability that avoids frustration or loss of autonomy [26].

Technological heterogeneity limits understanding of how and why TEC interventions succeed or fail in practice. As an umbrella term encompassing diverse tools and applications, TEC poses a significant methodological challenge for examining acceptance and impact within a single, unified framework. Nevertheless, successful development and implementation of TEC require a comprehensive understanding of the economic, social, structural, and technological barriers and facilitators that shape user engagement, including considerations of user-friendliness, accessibility, affordability, and ethical and regulatory constraints [27]. Accordingly, this rapid review scopes the literature to identify the multifaceted factors influencing TEC acceptability and its subsequent impact on the wellbeing of community-dwelling older adults. The findings will inform an initial framework to support the design, development, and implementation of TEC at scale. Specifically, the review addresses two questions: (a) What factors influence the acceptance, adoption, and sustained use of TEC by older adults and their caregivers in home settings? (b) What are the perceived and/or measured impacts of TEC on older adults’ lives at home?

## Methods and materials

Following the Cochrane Rapid Reviews Methods Group (RRMG) guidance, we employed a rapid review methodology to streamline the literature review process while maintaining rigour and evidence quality [28]. A rapid review methodology was deemed appropriate given TEC is broad and rapidly evolving area with direct relevance to service design, policy and implementation. Such an approach enabled timely synthesis of evidence across diverse technologies and study designs while retaining systematic and transparent process for searching, screening, charting and synthesis. We searched, screened and synthesised primary studies that investigated the acceptability and impact of TEC among community-dwelling older adults. The PRISMA 2020 checklist was used to present the result of the rapid review (see S1 PRISMA checklist) [29,30].

### Eligibility

Inclusion and exclusion criteria were developed based on the participants, concept, and context framework by the Joanna Briggs Institute [31]. Primary studies that investigated perspectives from older adults aged 55 and above, their caregivers, both informal (family or relatives) and formal caregivers, and other stakeholders (policy makers, social workers, community nurses, etc) were included. To inform the review questions, we retrieved studies addressing both the factors that influence the acceptance, adoption, and use of TEC among community-dwelling older adults and its impact. Studies involving clinical settings, telehealth, and remote health consultation were excluded from this review. Although these technologies fall within broad categories of TEC, they were excluded to maintain focus on technologies embedded in home and community living that support ageing in place, safety, monitoring, independence, communication and care coordination. Remote clinical consultation was considered outside the scope because they primarily involve clinician-led healthcare encounters rather than ongoing technology-supported living or care within the home environment. Peer-reviewed original primary studies published in English without geographical and methodological restrictions. Qualitative, quantitative, mixed-methods, feasibility, usability, implementation, observational, and intervention studies were included, provided that they reported primary data relevant to TEC acceptance, adoption, use, or impact. Reviews, protocols, editorials, opinion pieces, conference abstracts, and unpublished works were excluded (Box 1).

**Box 1. Eligibility criteria for the rapid review.**

**Inclusion criteria**

- Population: primary studies investigating population group of older adults 55 years or above, their caregivers, and other stakeholders (GPs, Health care providers, social workers, policy makers).

- Period: 2015–2025

- Concept: studies focussing on acceptance, adoption, use, impact of TEC

- Study design: Qualitative, quantitative, and mixed method studies

- Published peer-reviewed journal articles

- No geographical restrictions

- Studies published in English.

**Exclusion criteria**

- TEC types focussing on telemedicine and remote health consultations

- Study types: systematic reviews, conference papers, ongoing study protocols, editorial letters and opinions, and unpublished works

- Studies before 2015

- Language: any other language other than English

### Search strategy

Search strings and strategy combining the Population, Concept, and Context (PCC) elements were developed (Table 1). A high-yield sources database was prioritised: PubMed/MEDLINE, CINAHL, and Web of Science (S1 Text for the full search strategy of the databases).

**Table 1:** The PCC element, their definition and examples of search to be made for the rapid review.

PCC element	Definition	Example of terms
Population	Older adults who are 55 years old and above Studies involving OAs caregivers’ perspectives	“Older adults” OR “elderly” OR “aging population” OR “older people” OR “senior citizens” “carers” OR “caregivers” OR “family caregivers”
Concept	Technology-enabled care	“Technology-enabled care” OR telecare OR “mHealth” OR “remote monitoring” OR “assistive technology” OR “smart home technology” OR “health technology”
Context	Both community-dwelling OAs and those living in social housing	“Community dwelling older adults” OR “Social housing” OR “supported housing” OR “public housing” OR “affordable housing” OR “independent living” OR “community housing” OR “assisted living”

### Study selection process

All records identified through the search were uploaded to Covidence (Veritas Health Innovation, Melbourne, Australia), and duplicates were removed. A two-stage screening process was conducted. First, titles and abstracts were screened by AK, with a sample independently double screened by two additional reviewers (AMM and KC) to ensure consistency in the application of the eligibility criteria.

All studies meeting the eligibility criteria were considered for full-text assessment. Most studies excluded (*n* = 45) at full-text stage were excluded because they did not meet one or more eligibility criteria. In the second stage, the full-text of each eligible study was assessed by AK, AMM, and KC. Any discrepancies regarding inclusion were resolved through team discussion and consensus.

### Data charting and analysis

A standardised data extraction and charting tool in line with the rapid review questions was developed to extracts, key information, including author, year, research aims, technologies investigated, and main findings from studies that met the inclusion criteria. A descriptive analysis of the characterisations of the key findings was presented.

A multi-stage narrative and interpretive synthesis was undertaken to analyse findings across the included studies. First, data relating to Technology-Enabled Care (TEC) acceptance, adoption, and use were extracted and coded inductively, focussing on reported facilitators, barriers, and user experiences. These initial codes were iteratively grouped into conceptually similar categories, resulting in five overarching thematic domains: (1) individual characteristics and readiness, (2) perceived values and trade-offs, (3) sociocultural and relational dynamics, (4) contextual and structural enablers, and (5) technology design and appropriateness. These domains represent the primary descriptive synthesis of factors influencing TEC engagement. In a second analytic stage, an interpretive cross-cutting analysis was conducted to move beyond descriptive categorisation and identify higher-order mechanisms operating across these domains. This process involved examining patterns and recurrent explanatory concepts that appeared to shape how older adults interpreted and engaged with TEC across different contexts and technologies. These mechanisms were not treated as independent themes, but as interrelated processes that mediate the relationship between contextual factors and behavioural outcomes.

## Results

**Fig 1:**
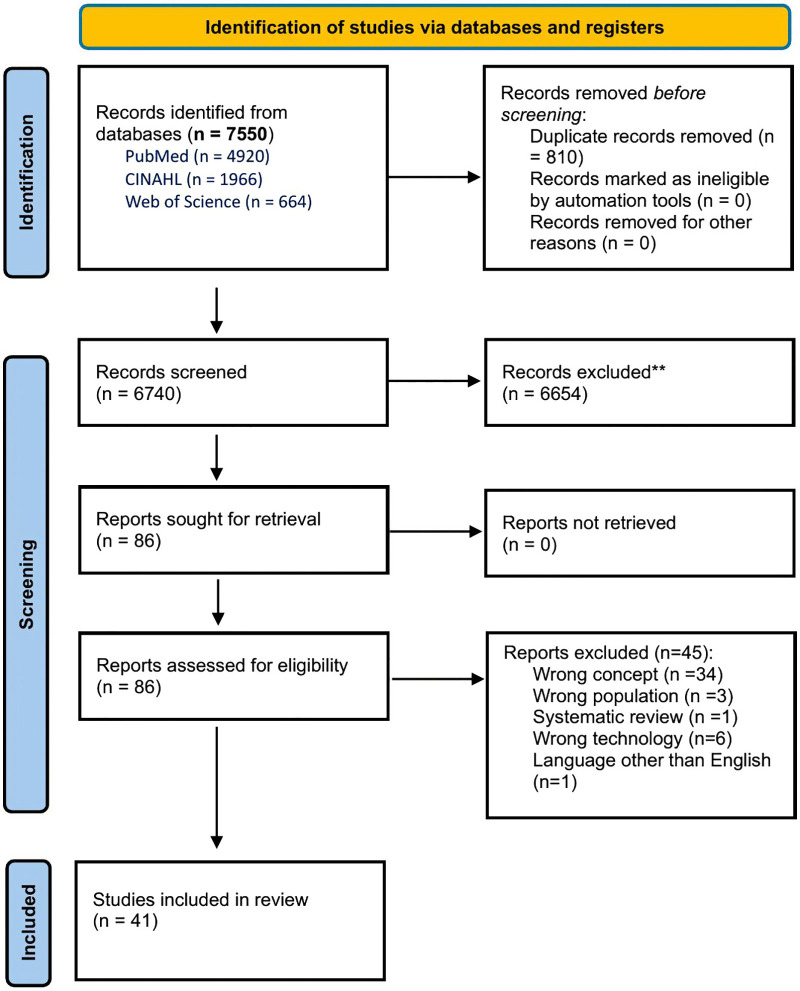
PRISMA Flow diagram depicting the study identification, screening, and included studies.

A total of 7550 articles were retrieved from the search on the three major databases PubMed (*n* = 4920), CINAHL (*n* = 1966) and Web of Science (*n* = 664). After removing 810 duplicates, the title and abstracts of 6740 studies were screened. Out of 86 studies found to be eligible for full-text review, 41 were included (See Fig 1 for PRISMA Flow chart).

### Characteristics of the included studies

Table 2 summarises the key characteristics of the included studies. More than 50% of the studies (*n* = 23) were published after 2020. Seven studies were published in 2016, which is the highest number in a single year. The majority of the studies originated from Europe, accounting for approximately half (*n* = 20), followed by North America (*n* = 9), Oceania (*n* = 7), and Asia (*n* = 5). The majority of the studies included in the review employed qualitative research methodology (50%). The majority of the studies (31 studies) investigated acceptance and adoption of TEC, while 10 studies investigated impact. Of the impact studies, five also examined aspects of acceptance/adoption; however, for the purpose of this review, studies were categorised according to their primary focus.

**Table 2:** Characteristics of the studies included in the study (n = 41).

Characteristics	Number (n)	Percentage (%)
**Countries**		
Europe	20	49
North America	9	22
Oceania	7	17
Asia	5	12
**Year published**		
2015–2019	18	44
2020–2025	23	56
**Study design**		
Qualitative	21	51
Quantitative	16	39
Mixed-methods	6	10
**Primary focus of the study**		
Acceptance/adoption	31	75.6
Impact	10	24.4

### Type of technology-enabled care investigated

The included primary studies investigated various types of TEC. This review organises the included interventions according to a typology based on interaction mode (Table 3). The majority of the studies investigated surveillance/safety TECs (*n* = 16) involving continuous monitoring and external oversight by caregivers or services, followed by passive monitoring technologies (*n* = 14), where technologies operate in the background with minimal user input. Furthermore, interactive support technologies requiring active user engagement for self-management and feedback (*n* = 3) and relational/shared care (*n* = 8), where technology use is embedded within social relationships and care networks.

**Table 3:** Type of technology in the included studies.

Interaction mode*	Definition/ Core characteristics	Examples	Core functions	# of Studies
Passive monitoring	Technologies that operate in the background with minimal or no active input from the user, collecting data automatically without requiring ongoing interaction. Low user engagement; “invisible” operation; embedded in home environment.	Ambient sensors; Smart home systems; Environmental monitoring	Risk detection, safety monitoring, automation, behavioural pattern tracking.	14
Surveillance & safety	Technologies that continuously monitor users and transmit data to external actors (e.g., caregivers, professionals, services) for oversight or intervention. Continuous data flow; external monitoring; perceived intrusiveness.	Telecare, remote monitoring, GPS tracking, in-home cameras.	Emergency response, health status monitoring, caregiver reassurance.	16
Interactive	Technologies that require active, intentional engagement from users to input data, interpret feedback, and manage their own care. High cognitive engagement; self-management; learning dependent.	Wearables, mobile health apps, digital health platforms.	Self-tracking, feedback, goal monitoring, health management.	3
Relational	Technologies whose function and meaning are mediated through social relationships and care networks rather than individual use alone. Social negotiation; caregiver mediation; identity sensitive.	PSADs, social robots, caregiver platforms.	Communication, care coordination, emotional and practical support.	8

* The categorisation in this table primarily groups studies by the overarching Technology-Enabled Care (TEC) investigated. We acknowledge the inherent overlap between technologies (e.g., remote monitoring is often integral to smart home systems), and thus an attempt to adhere closely to the authors’ descriptions was considered.

### Age-related findings through cohort and life-course mechanisms

Across the included studies, age-related patterns in TEC acceptance varied more by life-course stages, cohort exposure, how well they align with transitional circumstances and care trajectories than by chronological age alone. Lower TEC acceptance attributed to chronological age largely diminish once differences in education, usability, and facilitating conditions are taken into account. Quantitative studies included in this review demonstrated that once factors such as digital skills, prior technology exposure, perceived usefulness, and contextual supports were accounted for in the analytic model, the independent effect of age weakened or disappeared [32–36]. Though studies do not explicitly study these dynamics, the findings suggest that chronological age functions primarily as a proxy for cumulative digital exposure shaped by cohort-specific education, work histories, and infrastructural contexts, rather than intrinsic age-related limitations. Similarly, qualitative evidence reinforces that adoption is often triggered by key life-course transitions, such as retirement, onset of illness, falls, or hospitalisation, with uptake driven by perceived relevance to emerging needs rather than reluctance or resistance [37–40].

Sustained use was more strongly associated with integration into daily routines, perceived fit with care practices, and availability of social and professional support than with age or diagnosis per se [38,39,41,42]. Caregivers, family members, and health professionals played a central role in mediating adoption and meaning making, highlighting acceptance as a relational and intergenerational process [40,41,43]. Value-based concerns related to dignity, surveillance, autonomy, and identity further challenge age-based explanations, as resistance is often rooted in cohort-specific value systems shaped in pre-digital contexts rather than age-related incapacity [36,44,45]. Even among very old cohorts (≥80 years), studies reported meaningful benefits when technologies were relevant, well-designed, and supported, indicating that life-course exposure and support environments shape outcomes (Table 4) [38,46].

**Table 4:** Life course pathway of technology acceptance and use in later life.

Life-course stage	Late working life (55–65)	Transition to retirement & care (65–75)	High-support stage (75+)
Cohort exposure	Substantial workplace ICT exposure Formal education often includes computers Higher digital self-efficacy	Mixed digital exposure (limited workplace exposure) Uneven digital literacy Growing health variability	Minimal early digital exposure (No workplace ICT history) Strong pre-digital value systems Reliance on relational factors (others)
Typical technology exposure pathway	Technology first encountered at work, often mandatory	Technology re-introduced via health systems or family, not work	Technology introduced late in life, often after a crisis (e.g., fall)
Acceptance mechanisms	High perceived usefulness Ease of use less critical Strong capacity for customisation & personalisation	Acceptance driven by relevance to emerging needs (e.g., independence) Learning requires support Compatibility with routines critical	Acceptance highly contingent on trust and dignity Low tolerance for complexity (Simplicity and reassurance) Strong role of caregivers’ mediation
Technology type & use orientation	Mobile apps; Wearables; Digital health platforms; Monitoring & feedback tools	Home-based monitoring; Telecare; Cognitive support tools	Assistive technologies; Safety & alerting devices; Passive monitoring
Care orientation	Prevention & optimisation Self-management, productivity, risk reduction	Preparation & risk mitigation Maintaining independence, early decline management	Safety & care continuity Fall prevention, supervision, caregiver reassurance

### Acceptability of technology-enabled care

Primary studies exploring acceptability of TEC among older adults, their caregivers and other stakeholders were examined to identify factors influencing acceptance, adoption and continued use of these technologies (S1 Table for the detail of the studies). Five overarching themes of factors influencing acceptability of TEC (acceptance, adoption and use) were identified: individual characteristics and readiness, perceived values and trade-offs within technology, sociocultural and relational dynamics, contextual and structure enablers, and technology design and appropriateness. See Table 5 for summary of the factors and examples.

**Table 5:** Overall themes and factors influencing acceptance of TEC.

Themes	actors	Examples	Number of citations
1. Individual characteristics and readiness	Digital literacy and competency	- Awareness of technology existing and level of system understanding [50–52] - Comfort level [50] - Knowledge of system functions and limitations (e.g., how much data is collected, data is processed) [37,53,54] - Insufficient training [52] - Confidence and self-efficacy [43,52] - Technical skills [52]	7
	Health and psychological readiness	- Awareness of needs (understanding one’s situation necessitating technology) [59] - Health status and fall history [37,47,48] - Beliefs and concerns about physical capabilities or illness [43] - Emotional readiness and perceived need	5
	Experience and attitudes toward technology	- Past positive/negative experiences with technology [54,55] - Curiosity and willingness to explore [54,59] - Fear, stigma, or embarrassment (e.g., False alerts) [38,39,45]	6
2. Perceived values and trade-offs	Perceived usefulness and autonomy	- Safety net, accident prevention, alerting, independence [37–39,42,56] - Reminders and self-management of conditions [39,47] - Connection to healthcare [56]	6
	Concerns about privacy and intrusiveness	- Surveillance discomfort, security, data handling [37,39,44,50,54,57] - Concerns due to monitoring (e.g., uncertainty about when the camera was on, feeling watched) or false alerts [38,39,41] - Trust in system reliability and intentions [38,44]	8
	Symbolic meaning of technology	- Sign of dependence or decline [38,39] - Social image and stigma [43] - Identity and home space disruption [38,57]	4
3. Sociocultural and relational dynamics	Social influence and norms	- Influence of family, caregivers, friends, and community, pressure/support for adoption [40,42,47,51,58,59] - Primacy of social wellbeing [53] - Create new care network (e.g., pendant creating new care network for OAs living alone) [55] - Social stigma associated with some devices [43] - Framing by caregivers, health/social workers [41,44,45] - Direct involvement of community nurses in OAs care [40]	12
	Decision-making and consent	- Negotiation within families or with staff [40,44] - Visitors’ consent and shared living space dynamics [54]	3
	Cultural and language barriers	- Cultural expectations (e.g., independence vs. collectivism) [45] - Language barriers among minority groups [45]	1
4. Contextual and structural enablers	Access and affordability	- Cost of devices and maintenance [37,40,41,43,51,53] - Availability of financial or family support [59] - Stakeholder collaboration for scale up [63]	8
	Support systems and resources	- Coaching, personalised solutions [43] - Health and social care system support	1
	Infrastructure readiness	- Internet connectivity [47] - Integration into existing care models	1
5. Technology design and appropriateness	Technical functionality and reliability	- Accuracy, reliability, false alarms, GPS issues [39,45,47,50,53,58] - Sensor limitations and system flaws [43,48]	8
	Usability and comfort	- Device weight, design, compatibility with clothing [42] - Perceived ease of use and user interface [57,62]	3
	Fit with lifestyle and preferences	- Compatibility with daily routines [50,58] - Tailored to individual needs [43,51,63] - Aesthetic preferences (e.g., appearance, compatibility with the type of clothing worn, etc) [52,54]	7

#### A ) Individual characteristics and readiness.

Individual characteristics, including digital skills, health status, beliefs, sociodemographic factors and prior experiences, were consistently identified as key determinants of older adults’ acceptance of TEC. For example, lower educational status and older age were associated with lower acceptance [33], while perceived health status, beliefs about illness and perceived vulnerability were commonly cited as motivating factors for adoption, particularly for monitoring and emergency alerting technologies among individuals with a history of falls or chronic conditions [37,43,47,48]. Conversely, older adults who perceived themselves as relatively healthy often deferred adoption, anticipating a future need [49]. Acceptance was generally higher among those who perceived TEC as beneficial for managing health or alleviating wellbeing concerns, whereas scepticism and anxieties about potential negative impacts on care relationships (e.g., reduced in-person contact) acted as a barrier [39].

Digital literacy and self-efficacy emerged as salient individual characteristics influencing acceptance of TEC across multiple studies (*n* = 7). Higher levels of digital competence facilitated understanding of system functions, realistic expectations, and integration into daily routines, thereby reducing frustration and increasing perceived control [50–52]. In contrast, limited awareness, low understanding of system capabilities, and perceptions of complexity or irrelevance were cited as barriers to adoption [37,53,54]. Across studies, a reciprocal relationship was observed between digital competence, self-efficacy, and acceptance, with confidence predicting uptake and sustained use [43,52]. Emotional readiness and prior experiences further shaped attitudes, as positive experiences fostered curiosity and openness, whereas negative encounters (e.g., false alerts) reinforced apprehension and resistance [38,39,45,54,55].

#### B ) Perceived benefits, risks and symbolic meanings.

Older adults’ decision to accept and use TEC involved balancing perceived benefits against associated risks. Across studies, perceived usefulness of TEC as a safety net, particularly for accident prevention and fall detection, along with its potential to support independence and autonomy at home, were key facilitators of acceptance [37–39,42,56]. Technologies supporting daily routines, chronic conditions self-management, and communication with healthcare providers were viewed as valuable for maintaining control over health and everyday life [39,47,56]. However, concerns about privacy, intrusiveness, and surveillance emerged as major barriers. Continuous monitoring, particularly camera-based systems, generated discomfort and resistance due to fears of constant observation, data security, and loss of control over personal information [37,39,44,50,54,57]. Communication practices related to privacy, including phrases such as “I saw you went to the bathroom” or “I was watching you”, were perceived highly intrusive [44].

Symbolic meanings shaped acceptance, as some technologies were perceived to signal dependency, decline, or stigma, threatening older adults’ self-image and social identity [38,39,43,44,57]. For example, obtrusive device design (e.g., did not fit discreetly under clothing or does not match with the styles), false alerts, and embarrassment further undermined trust and willingness to use TEC [52,54]. TEC Acceptance reflects a nuanced trade-off in which perceived gains in safety, autonomy, and health management are weighed against risks to privacy, dignity, and identity, with trust in system reliability acting as a critical mediating factor [39].

#### C ) Sociocultural and relational dynamics.

Acceptance and use of TEC among older adults are deeply embedded within social relationships and cultural contexts. Across studies, adoption emerged as a relational process shaped by family members, caregivers, friends, health and social care professionals, and wider community or service networks [40,42,47,51,58,59]. Family and informal support were particularly evident in studies from Norway [39], New Zealand [53], in the Netherlands [59,60], in the UK [47], and China [61], where family members, informal networks, or integrational support influenced technology introduction, training, covering costs, confidence, and sustained use. Health and social care professionals also played a key mediating role, as the framing of TEC prior to installation strongly influenced older adults’ perceptions, with emphasis on independence and safety enhancing acceptance [41,44,45]. Negotiation within families and care relationships often determined whether and how technologies were adopted, highlighting the importance of addressing older adults’ preferences and concerns within shared decision-making processes [40,44,54].

Geographical and cultural context appeared to shape acceptance through different mechanisms across studies. For example, evidence from China [61] highlighted the role of intergenerational financial, emotional, and instrumental support in shaping intention to use smart home services, while studies from the USA [45] and Canada [51] showed how cultural expectations, acculturation, language barriers, and professional mediation influenced acceptance among immigrant, minority, or culturally diverse groups. Evidence from Finland also highlighted the importance of cultural adaptation, institutional trust, official advice, and social networks in shaping trust in digital assistive technology. These findings suggest that TEC acceptance was influenced by context-specific family roles, language needs, cultural expectations, and institutional or service arrangements, rather than by technology characteristics alone.

Cultural norms and values further shaped acceptance, particularly in relation to expectations around independence, intergenerational care, and the role of technology in replacing or supplementing human support. Technologies aligned with values of autonomy were more readily accepted in individualistic contexts, whereas concerns about erosion of human interaction were prominent in more collectivist cultures [45]. Language barriers and cultural mismatches also hindered understanding and effective use among minority groups [45]. Overall, the evidence highlights that TEC acceptance is not an individual or purely technical decision, but a socially negotiated and culturally mediated process embedded within networks of care and meaning.

#### D ) Contextual and structural enablers.

Acceptance and use of TEC among older adults are strongly shaped by broader environmental and systemic conditions. Across studies, access and affordability, particularly the cost of devices and ongoing maintenance, were consistently identified as major barriers, especially for older adults on fixed incomes [37,40,41,43,51,53]. Financial support from family or external sources was a key enabler allowing wider access to TEC and mitigating socioeconomic disparities in uptake [59]. The financial support extended beyond device purchase including, installation costs, subscription or monitoring fees maintenance, internet access, and access to ongoing technical support [34,39,53,62]. Without adequate resources, the potential benefits of TEC remain inaccessible to many who could benefit the most.

System-level support and infrastructure further influenced acceptance. The availability of tailored support, such as technology coaching and personalised solutions, enhanced confidence and facilitated sustained use, whereas lack of appropriate support contributed to abandonment [43]. Acceptance was higher when TEC was actively promoted within existing care pathways, particularly when technologies were framed as complementary to, rather than replacements for, traditional care, and integrated into established workflows. Reliable digital infrastructure, particularly internet connectivity, was identified as a prerequisite for functional use, with poor connectivity severely limiting effectiveness [47]. These findings indicate that TEC acceptance is also shaped by enabling environments characterised by affordability, institutional support, and robust infrastructure that integrate technology into routine care.

#### E ) Technology design and appropriateness.

Technological factors related to usability, reliability, functionality, and alignment with older adults’ lives and capabilities were consistently identified as critical determinants of TEC acceptance and use. Technologies perceived as complex, cognitively demanding, or poorly suited to users’ needs acted as barriers, whereas simple interfaces and ease of use facilitated engagement, particularly among individuals with lower digital literacy or physical limitations [42,57,62]. Physical design characteristics, including comfort, weight, and compatibility with clothing, also shaped willingness to use devices consistently, with bulky or intrusive technologies often rejected [42].

Technical reliability emerged as a central mechanism underpinning trust and perceived value. Accurate data collection, dependable alerts, and system stability were essential for sustained use, while technical failures, such as false alarms or inaccurate tracking, generated frustration, alarm fatigue, and abandonment [39,45,47,50,53,58]. Beyond functionality, technological fit and personalisation were key, as technologies that integrated seamlessly into daily routines and were tailored to individual needs and preferences were more likely to be accepted and sustained over time [43,48,50,51,58,63]. Overall, these findings highlight that TEC acceptance depends not only on what technologies do, but on how well they are designed to be usable, reliable, and meaningfully embedded in older adults’ everyday lives.

### Mediating mechanisms influencing acceptance of technology-enabled care

The review identified key themes that influenced acceptance, which arise from the complex interplay of individual perceptions and characteristics, technological features and contextual factors. Across all themes, the analysis revealed underlying mediating mechanisms: trust, the latent tension between intrusiveness and autonomy, stigma and identity, and perceived control. These factors do not operate in isolation, but instead they interact dynamically, influencing older adults’ readiness and willingness to engage with new technological solutions. See S2 Table for a summary of the underlying factors and their interconnectedness with the identified themes.

### Trust in technology

Studies have consistently highlighted trust as a foundational element in technology acceptance [50,64,65]. One study, for instance, demonstrated that the initial formation of trust in technology adoption and use is a complex interplay of multi-layered factors [65]. The study included an initial trust formation model developed by Lie et al (2008), which involved personal, cognitive, calculative, and institutional trust formation types. Factors such as human nature, the functionality and usefulness of the technology, familiarity, the reputation of the technology, and the roles and structures of the individuals were saliant. For older adults, this encompasses confidence in the technology’s reliability, accuracy, and data security. Specifically, concerns about false alerts, system reliability, and the privacy of personal data (e.g., from monitoring devices) can significantly erode this trust [54,64,66]. Conversely, trust is built through a transparent and dependable interface, along with reliable health and social systems that provide proper training, implementation, and oversight. In essence, trust in technology for older adults involves trust in the technology itself (its reliability and accuracy), trust in one’s own skills and self-efficacy to use it, and trust in the individuals introducing, handling, supporting, and implementing the technology.

### Intrusiveness versus autonomy

Studies have reported the latent tension between the perceived intrusiveness of technology and an older adult’s wish for autonomy [38,44,45,64]. Many TEC solutions, particularly those involving surveillance or constant monitoring (e.g., visible cameras, tracking devices), triggered feelings of being watched or controlled [39]. While such monitoring might be intended to enhance safety and enable independence by providing a ‘safety net’, paradoxically create emotional tension from invasion of privacy and a loss of personal space. The findings highlighted the challenge in designing and communicating technology that balances the benefits with the user experience and interest.

### Stigma and identity concerns

The impact of technology on an older adult’s stigma and identity was considered another cross-cutting theme. Some technologies, especially those that are highly visible or explicitly designed for specific age group (e.g., older adults’) populations were perceived as symbols of dependence or decline. Such fear of being labelled “old,” “weak,” or “dependent,” was reported to prompt resistance [38]. Aesthetic design that fit seamlessly into older adults’ daily life or clothing has the potential to reduce the identity conflict and obtrusiveness [52]. TEC has the potential to empower older adults to continue participation and maintains dignity, which is another form of challenging the negative stereotypes and narratives through technology.

### Perceived control

Older adults’ confidence in their skills and ability to use the technology (self-efficacy), understanding of its functions and limitations, and their ability to make informed choices about its use were considered as an important perceived control factor. Personalised training, customisable features, intuitive design, and flexible settings were cited as crucial. When older adults feel empowered to choose TEC that aligns with their needs, and easily navigate and manage the technology, it strengthens their sense of agency and leads to greater satisfaction and consistent use. Conversely, a lack of control, often due to complex interfaces or insufficient support leads to frustration and abandonment [51,59].

### Impact of TEC

Few studies (*n* = 10) investigated the impact of TEC on different aspects of older adults’ life and wellbeing (S1 Table for the details of the studies). These studies reported a measured or perceived impact of adopting and using TEC at home. Among these, 5 studies reported measured impacts using various measurement techniques, including quality of life measured using Visual Analogue Scale (VAS), quality of life assessed on 5 and 8 dimensions, the Personal Wellbeing Index (PWI), Canadian Occupational Performance Measure (COPM), Adult Social Care Outcomes Toolkit (ASCOT), and cost.

### Enhanced safety and independence

TEC contributed to older adults’ ability to live more independently and experience a higher quality of life. TEC acts as a safety net, offering features like fall detection and timely alerts that foster a sense of security and allow older adults to feel safer in their own homes [38,39,43,67]. This assurance extends to daily routines, where reminders support self-management and help individuals maintain control over their health and activities [39]. The positive influence on perceived daily life, social care-related quality of life, and a greater sense of independence collectively underscores TEC’s role in empowering older adults to achieve personal goals and improve their overall wellbeing and occupational performance [46,49,67,68]. This increased autonomy also indirectly alleviate the burden on family caregivers, further enhancing the overall living experience [49].

### Strengthening communication and social connection

TEC improves communication channels for older adults, both with their personal networks and with their care providers. The technology facilitates better communication with family and friends addressing and reducing feelings of isolation while enhancing vital social support [43]. This connectivity is not limited to personal relationships; TEC also streamlines communication between professional caregivers, healthcare providers, social workers, and the older adults themselves. Such improved connectedness and coordination ensure that all care is integrated, person-centred and responsive.

### Promoting health and prevention of adverse events

A key impact of TEC lies in its potential to actively contribute to the health and safety of older adults. Through features like timely alerts and monitoring, TEC enhances overall safety and helps prevent accidents such as falls. The ability of TEC to support self-management of chronic conditions empowers older adults to take a more active role in their health [43]. Moreover, some studies even suggested a direct improvement in neurological status, indicating TEC’s broader health benefits [69]. This proactive approach to health management and prevention not only safeguards older adults but also reduced reliance on more intensive and costly interventions.

### Driving efficiency and cost of health care

Beyond the direct benefits to older adults, studies highlighted that TEC demonstrates a promising potential for cost reduction within the healthcare system. One study specifically indicated that an intervention group utilising TEC experienced lower costs related to custodial care, emergency department services, inpatient stays, and overall healthcare expenditures when compared to a control group [70]. Accordingly, TEC has the potential to empower older adults by supporting safer and more independent ageing in place, while also reducing reliance on more expensive, institutional and acute medical services. This economic efficiency underscores TEC’s value not only as a tool for improving individual lives but also as a strategy for creating a more sustainable and cost-effective long-term care landscape.

### Adverse impacts and concerns

Despite TEC’s numerous benefits, there are challenges and potential adverse impacts, which also have implication on adoption and use. One of the adverse impacts cited was the psychological impact from the intrusiveness nature of technology. Particularly, camera surveillance technologies ensued a fear among older adults and their families ensuing from being constantly monitored [39,50]. Inaccuracies in GPS tracking devices also contribute to such anxieties, raising questions about data security and personal autonomy within the home.

Beyond privacy, users frequently encounter technical issues and difficulties, ranging from software glitches and hardware malfunctions to the ongoing need for maintenance. These problems were described to lead to frustration and diminish confidence in the technology’s reliability [39]. Furthermore, there is a risk of fostering dependency, where older adults feared developing an over-reliance on TEC, potentially diminishing the sense of self-efficacy or limiting their engagement in activities they might otherwise perform independently [38,39].

Paradoxically, while TEC aims to reduce caregiver burden, primary studies showed there are times where it increases responsibilities for family caregivers. For example, caregivers taking on the additional tasks of managing, troubleshooting, and maintaining the technology, adding a layer of complexity to their existing care duties [39,46]. As well as receiving alerts at night were discussed [45]. These multifaceted challenges highlight the importance of careful consideration of design, implementation, and support strategies to mitigate potential negative outcomes.

### Different measurement techniques of impacts of technologies on different outcomes

The majority of the studies reporting investigating impact of technology-enabled care (6/10) employed quantitative research method, while 2/10 utilised mixed method research. Various potential impacts of TEC were investigated, including cost, quality of life, wellbeing, achievement of personal goals. Different measuring techniques were reported, such as PWI, Quality of Life (Australian QoL), Goal Attainment Scale (GAS), Visual Analogue Scale (VAS). See Table 6 below for the description of the measurement methods and tools used with the aimed outcome measured.

**Table 6:** Different measurement techniques and outcomes demonstrating the impacts of technology-enabled care.

Measurement tool/method used	Outcome measured	Description of the method
Per-member, per-month (PMPM) cost data [70]	Lower mean total healthcare costs	Calculation of the average cost per member per month for healthcare services, allowing for a direct comparison of overall healthcare expenditures between groups.
Personal Wellbeing Index (PWI) [43]	Improved wellbeing (future security, achieving in life, other domains)	A self-report questionnaire where individuals rate their satisfaction with various life domains. Scores are compared pre- and post-intervention to assess changes in subjective wellbeing.
Australia QoL-8d (quality of Life-8D) [43]	Improved quality of life	A questionnaire specifically designed for the Australian context to measure perceived quality of life across several domains including independent living, self-worth, relationships.
COPM (Canadian Occupational Performance Measure) goals [43]	Improved goal performance, improved goal satisfaction	A client-centred outcome measure where individuals identify and rate the importance of everyday occupations they want to perform and then rate their performance and satisfaction with those occupations/progress toward personal goals.
Neuropsychological assessments [69]	Improved neuropsychological assessments	Standardised tests and evaluations designed to measure various cognitive functions (e.g., memory, attention, executive function).
ASCOT score (adult social care outcomes toolkit) [71]	Higher social care-related quality of life	A preference-based measure used to assess the quality of life specifically related to social care services. Higher scores indicate better outcomes.
Goal Attainment Scale (GAS) [46]	Achievement of personal goals	A client-centred measure where individualised goals are set with the participant, and progress towards these goals is evaluated on a standardised scale (e.g., a 5-point scale from much less than expected to much more than expected).
34 ADL items evaluated on three occasions [46]	Improvement in ADL items	A checklist or assessment of activities of daily living, where performance on 34 specific daily tasks to observe changes in functional independence.
VAS (Visual Analogue Scale) [46]	Improved self-perceived quality of life	A psychometric scale, where individuals mark a point between two extreme descriptors (e.g., “worst imaginable QoL” and “best imaginable QoL”) to indicate their subjective perception of their quality of life.
Zarit caregiver stress measures [46]	The level of stress from providing care to OAs	Implies the use of a questionnaire or scale designed to assess the level of stress experienced by caregivers.

### Integrative synthesis

**Fig 2:**
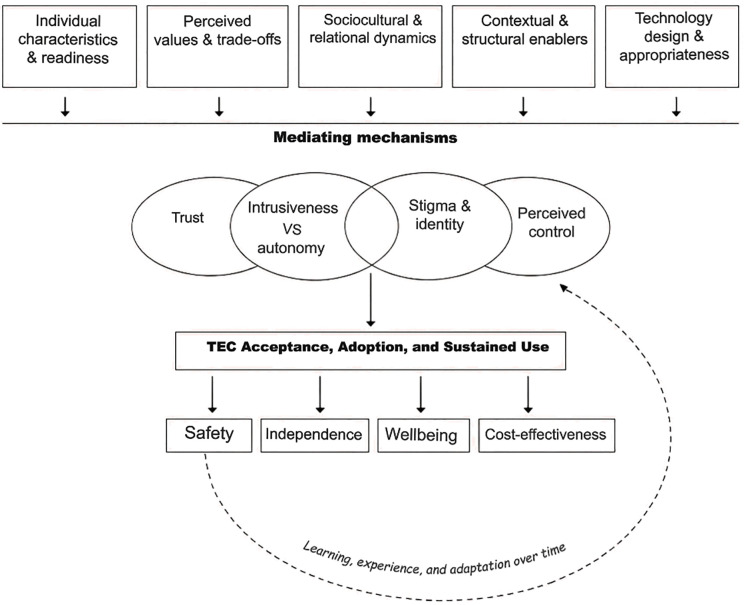
Framework of factors influencing TEC adoption and their mediating mechanisms.

Figure 2 presents an integrative synthesis of the main findings of this rapid review on TEC among community-dwelling older adults. The framework links the five thematic domains with four cross-cutting mediating mechanisms, trust, intrusiveness versus autonomy, stigma and identity, and perceived control, and maps these onto patterns of TEC acceptance, adoption, sustained use, and outcomes. The lower layer highlights key outcomes, including safety, independence, wellbeing, and cost-effectiveness, while the feedback loop illustrates the dynamic nature of TEC engagement, whereby experiential outcomes reshape mediating mechanisms and influence future use.

## Discussion

This rapid review synthesised evidence on the acceptance, adoption, and impact of TEC among community-dwelling older adults and demonstrates that engagement is shaped by interacting individual, relational, technological, and structural determinants rather than by single demographic predictors. Across included studies acceptance emerged not as a discrete decision but as a dynamic process mediated by trust, perceived control, identity and associated stigma, and the ongoing negotiation between autonomy and intrusiveness. These mechanisms influence whether technologies are initially accepted, meaningfully integrated into daily routines and sustained over time.

In particular, trust and perceived control emerged as central mechanisms shaping willingness to adopt TEC. Studies highlighted that older adults’ confidence in the reliability and safety of these technologies influences adoption readiness [72,73]. The balance between intrusiveness and autonomy is critical, as technologies designed to enhance safety simultaneously generate concerns regarding surveillance, privacy, and loss of independence. Evidence suggests that intrusive technologies may be accepted where perceived usefulness and independence benefits outweigh risks, whereas technologies perceived as stigmatising or threatening identity hinder acceptance [60,74]. Perceived value, anticipated quality of life improvements, and confidence in learning the system further contribute to adoption decisions [72,73].

Although older adults are often assumed to resist technologies, evidence indicates that attitudes towards TEC are generally positive [75]. The present findings suggest that perceptions of TEC depend not only on technical functionality but also on how solutions align with everyday routines, preferences, and sociocultural contexts. This was in line with a previous review, which identified perceived benefits, usability, affordability, social support, and individual characteristics as salient determinants of older adults’ acceptance and use of TEC [60]. These patterns are broadly consistent with established behavioural models such as the Technology Acceptance Model (TAM), and the Unified Theory of Acceptance and Use of Technology (UTAUT), which emphasise perceived usefulness, ease of use, and social influence as predictors of behavioural intention [19,76]. However, the present synthesis indicates that later-life TEC adoption additionally involves identity preservation, relational mediation, and autonomy negotiation, which extend beyond classical acceptance constructs.

This review also adopted an interpretive approach to examine patterns of TEC acceptance across life-course stages [77]. Although chronological age is frequently treated as a predictor of TEC adoption and age-related changes in sensory, cognitive, and functional changes, the evidence suggests that age largely functions as a proxy for differences in cumulative digital exposure, educational opportunity, and prior technology use. From a life-course and age-period-cohort perspective, readiness for TEC engagement is shaped by the timing of technology introduction and its alignment with transitions in health and care needs, as well as the unique values and preferences shaped by an individual’s life experiences [78,79]. Earlier exposure through work or everyday digital practices was associated with higher self-efficacy and smoother integration into routines, whereas crisis-driven late-life introduction typically required greater mediation from caregivers and services [80,81]. These findings suggest that proactive engagement with digital technologies earlier in the life course may widen opportunities for learning, habit formation, and long-term adoption, highlighting the limitations of using chronological age alone to explain technology engagement [82,83].

Substantial heterogeneity exists among older adults in terms of digital literacy, functional capability, health status, and attitudes toward technology. This variability is not merely descriptive but represents a salient determinant for the design, implementation and scale-up of TEC solutions. Accordingly, these findings reinforce the importance of adopting a robust human-centred approach grounded in co-production, where older adults’ and caregivers, and wider care networks actively involved throughout the technology development lifecycle, ensuring that systems are not only technically sound but also usable, meaningful, and aligned with older adults’ lived experiences [22,84–87]. Embedding tailored training and ongoing support within implementation pathways is similarly essential, as facilitation and user confidence play a central role in maximising both acceptance and the downstream impact of TEC on quality of life [88].

These user-level considerations translate into broader design and implementation challenges spanning both functional (what the system does) and non-functional (how the system performs) requirements. Functional priorities include truly intuitive interfaces and accessibility, while non-functional concerns including privacy protection, data ownership, and cybersecurity. Previous studies have identified persistent implementation barriers including limited methodologies for assessing digital literacy, the continuing digital divide, and insufficient integration of caregiver perspectives [89]. As current implementation efforts are limited by inconsistent approaches to assessing digital literacy. Many studies rely on broad indicators such as prior technology use, education, or self-reported confidence, which may not fully capture older adults’ practical ability to install, interpret, troubleshoot, and integrate TEC into everyday routines. Caregiver perspectives are also essential because caregivers often introduce technologies, provide training, respond to alerts, manage maintenance, and mediate decisions about privacy, safety, and continued use. Addressing these challenges requires interdisciplinary collaboration across engineering, health, and social care domains.

The technological landscape both research and practice in which TEC operates has evolved substantially. TEC has undergone significant evolution from personal alarms and analogue systems, to personalised, integrated services enabled by IOT, cloud analytics, and APIs. As countries increasingly implement digital transformation initiatives and switchovers, the demands on technical skills, structural and infrastructure readiness, cost and funding, and essential quality assurance measures posit challenges. Our review reflects some of the critical emerging concerns around privacy, autonomy, trust and other ethical issues. Although these considerations have always been present as a pretext, previous research such as that by Chen 2017, priorities usability and ease of use as the primary factors influencing acceptance [90]. This shift in focus is likely attributed to several key developments, including advances in pervasive data collection, the implementation of GDPR in Europe in 2018, and numerous high profile data breach incidents [91,92].

Alongside these developments, interoperability between TEC systems has emerged as a critical requirement. Historically, TEC solutions often existed as isolated services, also confined either to the home environment or community settings. There is a need for solutions that seamlessly communicate and share data. The data and insights generated through such connected systems are vital for health and social care markets to improve efficiency and cost-effectiveness, identify high-risk populations to trigger timely response and preventative interventions and allow older adults to age in place as long as possible.

When effectively implemented, TEC demonstrates substantial potential to enhance older adults’ wellbeing, particularly through improved safety, independence, and social connectivity. However, concerns regarding stigma, surveillance, and loss of control may undermine these benefits and hinder adoption, reinforcing the importance of respectful, culturally sensitive deployment strategies. Environmental and infrastructural conditions also play a foundational role. Reliable connectivity, affordability, and access to ongoing technical support remain structural prerequisites for meaningful engagement. Without addressing these system-level enablers, digital care expansion risks reinforcing existing inequalities [93].

In summary, these findings suggest that policy and practice should prioritise participatory design processes, sustained investment in infrastructure, and structured support mechanisms that strengthen digital literacy and user confidence. Ensuring robust privacy protections, ethical governance, and equitable access will be critical to enabling TEC to deliver benefits across diverse ageing populations.

### Strength and limitation

This study leverages a rapid review methodology, a highly efficient approach for synthesising evidence by streamlining the literature review process enabling quickly informing designers, policymakers, and other stakeholders about the complex landscape in which TEC is designed, developed, and implemented. By focussing on older adults’ acceptance of TEC, the multifaceted factors influencing it, and its impact on their lives, this review provides timely and actionable insights. Furthermore, we have included a substantial number of studies with a wide range of TEC investigated that reinforces the breadth of our evidence base.

While providing valuable insights this review has limitations that warrant considerations. First, the inherent nature of the methodology aims to directly address the research questions rather than to achieve exhaustive comprehensiveness. Accordingly, the number of databases selected for the review was limited to three (PubMed, CINAHL and Web of Science). Secondly, this study exclusively included published, peer-reviewed articles. Consequently, it may have missed valuable insights present in grey literature (e.g., conference proceedings, government reports, industry white papers, or pilot study reports). Such sources might offer different perspectives on engagement modalities, implementation strategies, and the acceptance and impact of TEC on community-dwelling older adults. Finally, the review primarily emphasised technologies employed within home settings. However, TEC is a broad and multifaceted domain. Our scope did not extensively cover technologies connecting clinical settings, residential care homes, or other diverse environments where older adults receive support or care. This focussed approach means the review’s findings may not fully capture the entire spectrum of TEC applications across all care settings.

### Conclusion and recommendations

There are multi-layered and complex factors to older adults’ acceptability of TEC and impact of TEC on their wellbeing and quality of life. Individual variations in digital literacy, physical capabilities, health status, and personal attitudes create a complex landscape that necessitates a departure from ‘*one size fits all*’ solutions. Instead, a human-centred approach, deeply rooted in co-production, is vital. This involves actively engaging older adults and their care networks throughout the TEC development lifecycle ensuring usable and useful TEC.

Design considerations are paramount for successful TEC implementation. Our review underscores the critical need for solutions that prioritise usability and accessibility, accommodating a spectrum of cognitive and physical abilities of community-dwelling older adults’. This includes features like simple user interfaces, comfortable and unobtrusive designs, and compatibility with daily routines and clothing. Furthermore, TEC must demonstrate robust technical functionality and reliability, as issues such as false alarms or inaccuracies erode trust and lead to abandonment. The ability of TEC being seamlessly integrated into older adults’ lives with personalised features is key to sustained use, as generic solutions often fail to meet specific needs.

Beyond design, mediating mechanisms, including trust, the tension between intrusiveness and autonomy, issues of stigma and identity, and perceived control, significantly influence TEC acceptability. Trust in technology’s reliability, accuracy, and data security is foundational, while perceived intrusiveness can paradoxically undermine the autonomy TEC aims to enhance. Designs that minimise stigma and empower older adults to maintain their identity are essential. Ultimately, fostering a sense of perceived control through intuitive design, customisable features, and adequate support is strongly linked to user satisfaction and consistent engagement. These insights underscore that successful integration and scaling of TEC demand a comprehensive strategy that prioritises ethical considerations, privacy protections, equitable access, and robust digital infrastructure.

## Supporting information

S1 PRISMA Checklist PRISMA 2020 checklist. (DOCX)

S1 Text Search strategies. (DOCX)

S1 Table Extracted characteristics of the studies. (DOCX)

S2 Table Summary matrix of thematic domains and cross-cutting themes. (DOCX)
