# Supplementary material for: Acceptance and impact of Technology-Enabled Care (TEC) among community-dwelling older adults: A rapid review
Source: PLOS Digit Health. 2026 Aug 4;5(8):e0001625. doi: 10.1371/journal.pdig.0001625 (PMC13436759; doi:10.1371/journal.pdig.0001625)
Supplement: Supplementary file 2 [file pdig.0001625.s002.docx]

**S1_Text**

**Search strategy for MEDLINE/PUBMED Database**

1. exp aged/
2. “older people”
3. “older person”
4. “older user”
5. “older adult*”
6. Ag*ing OR Elderl* or Geriatric or "Oldest Old" or "Older user" or "senior citizen*" or senior or nonagenarian or centenarian or "frail elder*" or Octogenarian?)
7. Exp telecare
8. assisted living.
9. remote care
10. digital health
11. assistive technology
12. (“Remote monitoring” OR "Remote Sensing"[Mesh] OR "Telemedicine"[Mesh] OR "Home Telemonitoring"[Mesh] OR "Remote Patient Monitoring" OR "Wearable Sensors" OR "mHealth" OR "Digital Health")
13. **“Ambient-Assisted Living" OR "AAL" OR "Smart Home" OR "assistive technology" OR "home healthcare technology" OR "homecare technology" OR "Technology-Enabled Care" OR "remote monitoring" OR "Digital health solutions" OR "Remote patient monitoring" OR "Home telecare" OR "Smart home technology"**
14. 1 OR 2 OR 3 OR 4 OR 5 OR 6
15. 7 OR 8 OR 9 OR 10 OR 11 OR 12 OR 13 OR 14
16. 13 AND 14

**Database(s): CINAHL Plus**

S9 S7 AND S8

S8 S4 OR S5 OR S6

S7 S1 OR S2 OR S3

S6 TI "Remote monitoring" OR "Telemedicine"[Mesh] OR "Home Telemonitoring" OR "Remote Patient Monitoring" OR "Wearable Sensors" OR "mHealth" OR "Ambient-Assisted Living" OR "AAL" OR "Smart Home" OR "assistive technology" OR "home healthcare technology" OR "homecare technology" OR "Technology-Enabled Care" OR "remote monitoring" OR "Digital health solutions" OR "Remote patient monitoring" OR "Home telecare" OR "Smart home technology" OR “fall detection*”

S5 "nursing informatics" OR "medical informatics" OR "consumer health”

S4 "Remote monitoring" OR "Telemedicine"[Mesh] OR "Home Telemonitoring" OR "Remote Patient Monitoring" OR "Wearable Sensors" OR "mHealth" OR "Ambient-Assisted Living" OR "AAL" OR "Smart Home" OR "assistive technology" OR "home healthcare technology" OR "homecare technology" OR "Technology-Enabled Care" OR "remote monitoring" OR "Digital health solutions" OR "Remote patient monitoring" OR "Home telecare" OR "Smart home technology" OR “fall detection*”

S3 TI "Older people" OR Aged OR "Aged, 80 and Over" OR "Frail elderly"

S2 AB "Older people" OR Aging OR Aged OR "Aged, 80 and Over" OR "Frail elderly" OR "Aged, Hospitalised"

S1 "Older person" OR "Older adult*" OR "Older people" OR Elderl* OR Geriatric OR "Oldest Old" OR "Older user" OR "senior citizen*" OR senior OR nonagenarian OR centenarian OR "frail elder*" OR Octogenarian?

**Database: Web of Science**

#1 TS=("Older people" OR Aged OR "Aged, 80 and Over" OR "Frail elderly" OR "Aged, Hospitalised") *Databases= WOS, BCI, KJD, MEDLINE, RSCI, SCIELO Timespan =All years*

#2	TI= ("Older people" OR Aged OR "Aged, 80 and Over" OR "Frail elderly")

#3	AB=("Older people" OR Aged OR "Aged, 80 and Over" OR "Frail elderly" OR "Aged, Hospitalised") *Databases= WOS, BCI, KJD, MEDLINE, RSCI, SCIELO Timespan=All years*

#4 TS= ("Remote monitoring" OR "Telemedicine"[Mesh] OR "Home Telemonitoring" OR "Remote Patient Monitoring" OR "Wearable Sensors" OR "mHealth" OR "Ambient-Assisted Living" OR "AAL" OR "Smart Home" OR "assistive technology" OR "home healthcare technology" OR "homecare technology" OR "Technology-Enabled Care" OR "remote monitoring" OR "Digital health solutions" OR "Remote patient monitoring" OR "Home telecare" OR "Smart home technology" OR “fall detection*”)

#5 TI=("Remote monitoring" OR "Telemedicine"[Mesh] OR "Home Telemonitoring" OR "Remote Patient Monitoring" OR "Wearable Sensors" OR "mHealth" OR "Ambient-Assisted Living" OR "AAL" OR "Smart Home" OR "assistive technology" OR "home healthcare technology" OR "homecare technology" OR "Technology-Enabled Care" OR "remote monitoring" OR "Digital health solutions" OR "Remote patient monitoring" OR "Home telecare" OR "Smart home technology" OR “fall detection*”)

#6 AB= ("Remote monitoring" OR "Telemedicine"[Mesh] OR "Home Telemonitoring" OR "Remote Patient Monitoring" OR "Wearable Sensors" OR "mHealth" OR "Ambient-Assisted Living" OR "AAL" OR "Smart Home" OR "assistive technology" OR "home healthcare technology" OR "homecare technology" OR "Technology-Enabled Care" OR "remote monitoring" OR "Digital health solutions" OR "Remote patient monitoring" OR "Home telecare" OR "Smart home technology" OR “fall detection*”)

#6 #1 OR #2 OR #3

#7 #4 OR #5 OR #6

#8 #6 AND #7
