# Supplementary material for: Acceptance and impact of Technology-Enabled Care (TEC) among community-dwelling older adults: A rapid review
Source: PLOS Digit Health. 2026 Aug 4;5(8):e0001625. doi: 10.1371/journal.pdig.0001625 (PMC13436759; doi:10.1371/journal.pdig.0001625)
Supplement: Supplementary file 3 [file pdig.0001625.s003.docx]

**S1 Table Characteristics of studies investigating acceptability of TEC (n=36)**

| **Author(s)** | **Study design** | **Country** | **Study population** | **Care setting/context** | **Type of TEC** | **TEC purpose/function** | **Review focus** | **Factors influencing acceptance, adoption and sustained use** |
| --- | --- | --- | --- | --- | --- | --- | --- | --- |
| Wang et al, 2024 | Quantitative | China | 55 years and older, 475 participants | Community/home context | Smart home | Smart home technology to support ageing in place, behavioural intention, and home-based support. | Acceptance/intention to use | Perceived usefulness (beta=0.17) and perceived ease of use (beta=0.103) directly affect behavioural intention and at the same time mediate social influence, perceived trust, compatibility. Perceived cost directly negatively affected behavioural intention to use SH (beta=-0.19*) |
| Karlsen, 2018 | Qualitative | Norway | 18 OAs 60 years and above and a follow up interview with 15 and 7 family caregivers; received telecare services (less than 3 months) | Home/community telecare services; older adults and family caregivers | Telecare | Telecare for safety, alerts, reminders, independence, and ageing at home. | Adoption and sustained use; impact | Family caregivers played an important role in adoption and sustained use of telecare technologies. Bought, installed telecare services. Knowledge about the possibilities and how to use the device was essential |
| Abolhassani,2019 | Quantitative | Switzerland | n=3195 community-dwelling 69–83-year-old members of the Lausanne cohort 65+ | Community-dwelling older adults | Assistive information technology: Electronic health records/cards Telecare | Health information and communication technologies, including telecare, alarms, sensors, and health records/cards. | Acceptance | Acceptance of health-related technologies -remote alarm as a bracelet higher among the age group 69-73 in both genders (70% vs 75% in men and women, respectively. Position sensors were 40% on average in both genders. Demographic Factors: Age: Older age was associated with rejection of HICTs (OR=1.54 (1.14-2.09) 79-83 compared to 69-73).    Education: lower education levels were also linked to lower acceptance. (OR=2.89 (2.07-4.02) compared to high education.    Gender: men were more likely to reject HICTs (OR=1.92)   Health-related factors: Difficulties in Activities of Daily Living (ADLs): participants with difficulties in ADLs were less likely to accept HICTs. OR=1.44(1.02-2.04) compared to those without basic nor in instrumental ADL difficulty |
| Arthanat, 2020 | Quantitative | USA | 454 older adults 65 years and above | Community/home context | Smart home | Smart home automation for ageing in place, safety, independence, and home security. | Adoption/ownership | ICT usability (0.15), home security (0.28) had a significant positive direct effect on SH ownership \|\| Self-reported Independence in daily routines negatively affected SH ownership (b=-0.23); home safety plays a mediating role between different factors and SH ownership. Physical impairment and incidents of falls and accidents have a negative effect on home safety (-0.84), which in turn influences smart home ownership. Social support has an indirect effect on smart home ownership, mediated through home security (0.1). Demographic variables age, gender, education were not significant |
| Berridge, 2016 | Qualitative | USA | 41 study participants; Including- 6 low-income independent living residence apartment buildings -- 20 older adults', emergency contacts, Staff (social workers and supervisors) | Low-income independent living/senior housing | Sensor based passive monitoring systems | Sensor-based passive monitoring for activity monitoring, alerts, and safety in independent living. | Acceptance/adoption | Adoption of this technology was very low and privacy and intrusiveness was major factor. Awareness that the technology doesn't collect visual image or it is not camera was mentioned as a factor facilitating adoption. Additionally social workers and how they explain the technology was found to improve acceptance. |
| Berridge, 2019 | Qualitative | USA | 41 study participants from a large senior housing independent living apartment buildings focuses on older adults from low income and immigrant population--Residents, family and staff | Low-income and immigrant senior housing | Sensor based passive monitoring systems | Sensor-based passive remote monitoring for movement/activity tracking and caregiver alerts. | Acceptance/use; cultural context | Cultural values & expectations: acceptance of monitoring is shaped by elders' beliefs about care. e..g., Russian elders preferred hands-on care, while some Asian elders accepted reluctantly due to filial piety. Acculturation: adapting to the host culture influences technology acceptance as immigrants balance their values with new US norms. Intergenerational conflict: family cultural differences, like views on caregiving roles, impact technology use. e.g., Asian elders discontinued use to avoid burdening children, highlighting conflicts with filial piety. Social workers as navigators: Multilingual social workers bridge cultural gaps, with their support crucial for technology adoption. Communication & language: language barriers hinder acceptance and effective use.e.g., language issues at call centers and during emergencies create challenges. Technology design & implementation: cultural sensitivity in design and service delivery is key for acceptance. e.g., lack of cultural awareness from technology companies can be a barrier. |
| Claes, 2015 | Quantitative | Belgium | 245 older adults 60 years and above | Community/home monitoring context | Contactless monitoring | Contactless monitoring of activities of daily living to support safety, independence, and timely detection of problems. | Acceptance/willingness to use | potential usefulness, availability of collected information, functional requirements and financial costs, and the use of video cameras; respondents or installers being able to respond to their question on monitoring; ability to temprarily switch off technologies \\unintelligible error messages (71.7%), ageing associated physical limitations including vision, hearing, manipulating small buttons (70.3%) or privacy (64.4%). A possible feeling of stigmatization (35.1%) or visibility for visitors (30.6%) were seen as minor concerns. False alarms, 70.5% would not want to pay maintainance cost, more than 90% want goverment or social security pays for it |
| Deguchi, 2024 | Quantitative | Japan | 899 older adults 65 years and above | Community-dwelling older adults | Smart Watches | Smartwatches for health, activity monitoring, and individual health management. | Acceptance/purchase intention | Smartwatch acceptance: In men, younger age <75 years (OR=2.1 (1.2-3.7), obesity, diabetes, and dyslipidemia were significantly associated with smartwatch acceptance. In women, younger age (OR=1.7(1.05-2.71), living alone (OR=1.98(1.23-3.18), higher household income, and a high score for new device use in the technology literacy category were significantly associated with smartwatch acceptance.    Purchase intentions: Among men, younger age, lower household income, obesity, diabetes, and dyslipidemia were associated with higher purchase intentions. Among women, living alone and high scores for new device use and gathering information were associated with higher purchase intentions. |
| Dermody, 2023 | Qualitative | Australia | 10 Family caregivers | Family caregiving/home context | Smart home technology | Health smart home technology to monitor care-dependent older adults and support family caregivers. | Caregiver readiness/acceptance | Caregivers readiness was influenced by: big brother effect(concerns about older adults feeling watched)    Framing for acceptance (how caregivers introduce the technology to older adults)    Data privacy    Burden(potential for reducing caregiver burden)    Cost |
| Dermody, 2021 | Qualitative | Australia | 19 community dwelling older adults' | Community/home context | Smart home technology | Smart home technology to support ageing in place, safety, independence, and quality of life. | Readiness to adopt | The following themes were important influencing older adults' readiness to adopt smart home technology:- Knowledge and understanding of the technology    Perceptions of health and safety    The desire to maintain independence and autonomy , Concerns about security and privacy ; Cost considerations |
| Elers,2018 | Qualitative | New Zeeland | 10 older adults and 31 self-identifies informal support networks | Home/community context with informal support networks | home monitoring technology and ICT | Home monitoring and ICT to support ageing in place, information sharing, and connection with support networks. | Acceptance/adoption | Social enablers- social wellbeing was priority to accept technology compared to medical condition, the ability to be connected with social groups, technology related concern and information desired (Knowledge of how much data is collected); cost of technology |
| Etemad-Sjadi, 2019 | Quantitative | Switzerland | 213 respondents with mean age of 82.1 | Community-dwelling older adults | Connected health technologies | Connected health technologies, including alarms, telecare, and sensors, to support home-based care. | Intention to accept | usefulness directly and positively affects intention to accept (0.64*); While trust (0.38*), social presence (0.18*), ease of use (0.45*) affect directly usefulness and usefulness act as a mediating factor for these factors. |
| Farina, 2018 | Mixed method | UK | 26 cares and 26 persons with dementia | Home/community dementia care context | Activity monitors | Wearable activity monitors to measure physical activity among people with dementia. | Acceptability/satisfaction | Satisfaction with technology was higher among person with dementia assessed: dimension (60%), weight (80%), ease in adjusting (82%), safe and secure (88%), durability (88%), comfort (60%) |
| Ghorayeb, 2021 | Qualitative | UK | 13 older adults between 66 and 89 | Home/smart home context | Smart home technology | Smart home monitoring technology for health and lifestyle monitoring in the home. | Acceptance/use | Among SH participants (with experience): More interaction with the system, aesthetic appeal, curiosity about the system how data is collected and decision of alarm is processed need for more information despite the initial introduction, making visitors aware (consent as monitors might not be turned off) \|\| similar acceptance factors between the groups: technical feasibility and affordability, impact on human relationships, competence and engagement of people monitoring the data; desire for customisation \|\| Differences - acceptance over time among shp, privacy and trust, Usability, utility among participant with experience, vulnerability and intrusiveness, |
| Gibson, 2023 | Qualitative | UK | 21 older adults intervered in three rounds of interviews pre-installation and after | Home context; pre- and post-installation interviews | Remote monitoring systems | Remote monitoring system to support safety, risk detection, and care connections in the home. | Acceptance and use over time | Pre-Installation: participants focused on the meaning of home, security, and their expectations of the monitoring device, contemplating its integration into their lives while weighing privacy concerns against potential safety and independence benefits. Installation: the installation process brought awareness to the technology's physical presence and implications for daily routines and privacy, with some finding it straightforward and others struggling with perceived intrusiveness, especially near intimate areas.    Post-Installation: reflections on the monitoring system varied, with some participants integrating it seamlessly and others maintaining discomfort, particularly regarding monitoring of intimate activities, and some participants reassessing their initial perceptions of intrusiveness over time. |
| Gordon, 2022 | Mixed method | Australia | 30 community dwelling older adults 65 and above with mean age of 79 years | Community/home context | Health monitoring, communication and entertainment devices and security alarms | Individualised technology package for health monitoring, communication, entertainment, security, and safety goals. | Acceptance/use; impact | Enablers:  Technology coaching  Individualized technology packages  Addressing specific needs and goals  Barriers:  Cost  Lack of confidence and low digital literacy  Physical limitations  Technology not meeting expectations  Social stigma associated with some devices (e.g., fall monitors) |
| Igarashi, 2023 | Quantitative | Japan | 400 community dwelling older adults 65 and above: this study included 200 older adults living alone and 200 couples | Community-dwelling older adults; living alone or as couples | Ambient sensors | Ambient sensors for household monitoring, safety, security, and daily-life support. | Acceptance/adoption; impact | Age was a significant factor for sensor adoption, over 75 willing to install sensors compared to 65-74; Percevied benefits of the technology, usefullness of the system, positive image of the technology, innovateveness of the individual. There is no difference of the attitudes towards introduction of sensor systems between the two groups. However, the study suggests that couples household have high activity capacity index, which tend to promote autonomous living support-- could reduce adoption of sensors. reduced expenses and increased income were highly important. |
| Kim, 2020 | Qualitative | USA | 20 community dwelling OAs with visual disabilities | Community/home context; older adults with visual disabilities | Sensor technology | Sensor technology for home activity monitoring and safety among older adults with visual disabilities. | Implementation/installation acceptance | Four main categories influence implementation and installation of sensors: People. Environments, Tasks, and Tools. For example, factors related to the person include emotional challenges, fall history, privacy concerns, and technology adoption preferences.  Environmental factors include home size, layout, presence of stairs, and potential obstacles.  Task-related factors involve activities of daily living, exercise habits, cooking, and walking patterns.  Tool-related factors consider the use of walking aids and the technical limitations of the sensor. |
| Jaschinski, 2021 | Quantitative | Netherland | 1296 older adults | Community-dwelling older adults | Ambient Assisted Living Technologies | Ambient assisted living technologies to support ageing in place, health, autonomy, and independence. | Intention to use | attitudes toward using AAL, social norms, personal norms, and perceived behavioral control significalntly influence intention to use AAL and negative factors include concerns about loss of privacy, loss of human touch, and financial costs. |
| Lie, 2016 | Qualitative | UK | 24 interviews in first trial and 43 interviews in the second trial with OAs | Home monitoring trial context | SHel home monitoring system. | Home monitoring system for safety, privacy-sensitive monitoring, and support for ageing in place. | Acceptability/use; impact | Safety and privacy are key themes in influencing acceptability. Trust was identified as a crucial factor underpinning acceptance. A tradeoff between safety and privacy. Pre-trial: Trust vs. Privacy: trade off between safety and privacy  Initial impressions: participants' first thoughts about the system influenced their willingness to accept it. Comfort level was affected by the level of system understanding  There were worries about data collection, access, confidentiality, and storage were factors.  Post-trial: Actual use of the system shaped whether it was seen as trustworthy and useful.  If the system increased the older person's sense of safety, it likely increased trust.  If the system fit well into the older person's routines and habits, it was more likely to be accepted.  Trust in the technology: system's reliability and accuracy influenced trust. Trust in monitors: the relationship between the older person and their monitor (often a family member) was crucial; trust in the monitor's intentions and handling of the data was key. |
| Peek, 2016 | Qualitative | Netherlands | 53 community dwellign older adults between the age of 68 and 95 | Community-dwelling older adults | Various types of technology | Various technologies to support independent living, ageing in place, and implementation in home/community care. | Acceptance/adoption | Challenges in the domain of independent living: encompassing the difficulties older adults face in maintaining their independence and how technology can assist with these challenges. Behavioral options: this theme refers to the choices older adults make regarding their behavior and lifestyle, and how technology fits into these choices. Personal thoughts on technology use: involves older adults' attitudes, beliefs, and perceptions about technology, which can significantly influence their willingness to use it. Influence of the social network: highlights the impact of social connections, such as family, friends, and community, on older adults' technology adoption and use. Influence of organizations: this theme considers the role of formal organizations, such as healthcare providers and technology companies, in promoting or hindering technology use among older adults. Role of the physical environment: This theme acknowledges the importance of the physical surroundings in which older adults live and how technology interacts with this environment. |
| Middlemass, 2017 | Qualitative | UK | 21 patients between the age of 60 and 99 years --people with history of COPD or heart failure and their caregivers | Home telemonitoring context; COPD or heart failure | Home telemonitoring | Home telemonitoring to monitor long-term conditions and support health-related self-management. | Acceptance/use | Health-related factors included patients' health status, health beliefs, and concerns about their illness. Some patients were concerned that telemonitoring might replace face-to-face contact with healthcare professionals.  Technology-related factors included HIT reliability (e.g., internet connectivity, accuracy of equipment), HIT self-efficacy (i.e., confidence in using the technology), perceived ease of use, and perceived usefulness.  Social factors also played a role, with support from healthcare professionals, family, and friends influencing patients' decisions to use telemonitoring. |
| Pais, 2020 | Quantitative, | Switzerland | 13 older adults, 13 family care givers, 20 nurses | Home care/in-home monitoring context; older adults, caregivers and nurses | in-home monitoring system | In-home monitoring system/sensors to support home care, safety, and quality of life. | Acceptability; impact | Opinions tended to be more favorable toward ambient sensors than wearable sensors.  Older adults and family caregivers tended to be more enthusiastic than nurses.  Barriers reported by nurses included fear of weakening their relationship with older adults, work overload, lack of time, and cumbersome procedures. |
| Peek, 2016 | Qualitative | Netherlands | 53 community dwellign older adults between the age of 68 and 95 | Community-dwelling older adults | Various types of technology | Various technologies to support independent living, ageing in place, and implementation in home/community care. | Acceptance/adoption | Challenges in the domain of independent living: encompassing the difficulties older adults face in maintaining their independence and how technology can assist with these challenges. Behavioral options: this theme refers to the choices older adults make regarding their behavior and lifestyle, and how technology fits into these choices. Personal thoughts on technology use: involves older adults' attitudes, beliefs, and perceptions about technology, which can significantly influence their willingness to use it. Influence of the social network: highlights the impact of social connections, such as family, friends, and community, on older adults' technology adoption and use. Influence of organizations: this theme considers the role of formal organizations, such as healthcare providers and technology companies, in promoting or hindering technology use among older adults. Role of the physical environment: This theme acknowledges the importance of the physical surroundings in which older adults live and how technology interacts with this environment. |
| Peek, 2017 | Qualitative | Netherlands | 33 community dwelling older adults | Community-dwelling older adults | various technologies | Various technologies acquired to support independent living and everyday needs. | Technology acquisition/adoption | older adults' needs, perceptions, and the influence of family and friends influence technology acquirment. Specific factors incluing awarness of needs (e.g., needs a ride at night, so gets a mobility solution), motivation to acquire (e.g., buys a tablet because it's cheap and looks good), resources (e.g., use savings to buy a device, family pays for a personal alarm) and the influence of social networks (e.g., gets a smartphone after seeing friends use it). |
| Peek, 2016 | Qualitative | Netherlands | 29 stakeholders including OAs(older adults (6), care professionals (7), managers within home care or social work organizations (5), technology designers and suppliers (6), and policy makers (5) | Community/home care stakeholder context | various types of technology | Various technologies to support independent living, ageing in place, and implementation in home/community care. | Implementation/adoption | Successful implementation require prioritizing older adults' needs and wishes, ensuring technology acceptance by older adults, providing benefits to older adults, and establishing favorable prerequisites for technology use (e.g., ease of use, affordability, reliability). tailoring technology to the specific needs of older adults, which implies that implementation steps should include a strong focus on individual assessment and customization. |
| Pol, 2016 | Qualitative | Netherlands | 11 older adults between the age of 68 and 93 | Home wireless sensor monitoring context | a wireless sensor monitoring system | Wireless sensor monitoring system for safety and professional access to home-based monitoring data. | Acceptance/use; impact | Privacy violation was a concern that influnced acceptance and use. Perceived benefits: the benefits of healthcare professionals' access to sensor data for safety outweighed privacy concerns. |
| Pitchard, 2015 | Qualitative | UK | 47 older adults (45 live in the community and 2 lived in a care facility) and 9 carers | Community/home context; includes two care-facility participants | Alarm pendant | Alarm pendant for emergency response and care-network support. | Use/acceptability | Social realtions influnece the functions of alarm pendant and vice versa: Influence of social relations on alarm pendant function: pendants support existing care networks in facilities but may establish new ones for older adults living alone. (e.g., in an extra-care facility, alarm pendants primarily support and enhance the existing care network. In contrast, for older adults living alone, the pendant might establish a new care network. )  Influence of alarm pendant function on social relations: alarm pendants reduces face-to-face contact and altering caregiving dynamics. This raises the risk of dehumanization, diminishing older adults' agency, and may unintentionally increase the workload for older adults and carers. |
| Read, 2022 | Qualitative | Canada | 28 interviews with different group of people (2 older adult patients; 11 caregivers, 8 social workers, 7 key government decision makers) | Home/community passive remote monitoring context | In-home passive remote monitoring technology | In-home passive remote monitoring service to support home care decision-making and caregiver awareness. | Awareness/recommendation/adoption | Lack of awareness and knowledge of the service.  The technology's fit with the needs of the client and their family or friend caregiver.  Social workers' perceptions of which clients they should recommend the technology to.  Preference for traditional or familiar home support services.  Concerns about privacy.  Costs Language |
| Sorwar, 2022 | Quantitative | Australia | 60 older adults between the age of 68 and 90 | Community/home smart home context | Smart Home Technology (SHT) | Smart home technology to support ageing in place, perceived usefulness, and adoption intention. | Acceptance/adoption | Factors Influencing Acceptance and Adoption: Perceived Usefulness (pre-use beta=0.308*, post beta=0.435*) cindluence behavioral intention Trust (found to indirectly predict adoption via Perceived Usefulness) (pre use beta=0.362* and post use= 0.495*)-- were the two significant variables |
| Reeder, 2020 | Qualitative | USA | 10 older women with average 64.6 years | Community/home context; older women | Wearable sensors | Wearable sensors for monitoring and supporting safety or health-related awareness. | Adoption/use | Factors include usability issues, lack of technology skills, insufficient training, privacy concerns, and low self-efficacy. Older adults may reject technologies if they don't understand the benefits. Aesthetic preferences influence adoption (e.g., appearance, incompatibility with the type of clothing worn, having something like bump under clothes) |
| Thilo, 2021 | Qualitative | Switzerland | 33 relatives (daughters, sons and husband), 34 community nurses, 11 GP | Community/home care context; relatives, nurses and GPs | Personal Safety Alerting Devices (PSADs) | Personal safety alerting devices for emergency alerts, safety, and negotiated home-care support. | Adoption/negotiated use | The negotiation process between older adults, relatives, and healthcare professionals, which includes: • Waiting for a critical event in the older adult's life. • Introducing the idea of a PSAD.  • Deciding on and supporting PSAD use.  Costs.  Social pressure.  The alerting process (how and to whom the alert is transmitted).  PSAD characteristics. |
| Tural, 2021 | Mixed method | USA | 129 surverys and 15 OAs participated in FGD | Community/home smart home context | Smart home technology | Smart home technologies, including safety and fire-prevention devices, for home safety and ageing in place. | Acceptance/willingness to use | Perceived usefullness predicts significnantly both attitudes and willingness to use SH products; Fire prevention devices had higher perceived beenfts however perceived affordability inlfunce(Perceived ease of use (OR=2.63 AND perceived usefullness (OR=7.84) significantly predcits better attitude toward smart fire prevention); technology skepticism, privacy concerns and return on investment (justifying purchase and installation, e.g. high upfront cost and installation fee ); digital divide such as technology access, skills |
| Mesthrige, 2022 | Quantitative | Hong Kong | 201 older adults who are either in public housing scheme or private sector housing | Housing context; public and private housing | Smart home technologies | Smart home technologies to support ageing in place in public and private housing contexts. | Barriers to adoption/acceptance | Three categories of critical barriers : (1) cost (Financial concerns include cost of acquiring, installing and maintaining) and knowledge barrier (0.778) this include high cost of maintenance, limited knowledge about the devices, devices not reliable (2) Personal concern (0.791) (Smart home will make me more lazy and inactive; Smart home will disrupt my desired daily routine; Smart home will make me to worry more; Smart home will increase dependence on outside experts; I can loss control of my home and (3) Uncertainty (0.797): Lack of contingency plan in the event of power failure; The fear of systems being compromised; I do not fully understand the functions and consequences of smart home; Smart home will not fit to my current and changing lifestyle : all factors greater than 0.5 factor loading |
| Weck, 2023 | Qualitative | Finland | 41 older adults | Community/home context | Digital assistive technology | Digital assistive technology to support independent living and care-related trust. | Trust/acceptance | Personality base of trust (positive trusting beliefs in human nature, functionality of technology, technological advances and helpfulness)  Cognitive base of trust (cognitive familiarity, reputation of technology, reliability of technology and service providers, human roles and structures)  Calculative base of trust (risk of exposure to opportunistic behaviour, positive beliefs in official advice)  Institutional base of trust (existence of "situational normality," structural assurance, control over personal information)  Supplementary factors (ICT self-efficacy, support of social networks, cultural adaptation, existence of the "digital divide") |
| Wei,2023 | Quantitative | China | 298 older adults | Community/home smart home context | Smart home technologies | Smart home services shaped by intergenerational support and perceived usefulness/ease of use. | Intention to use/adoption | Intergenerational relationships significantly influence the utilization of smart home services among older adults. Intergenerational financial support (0.308*) and Intergenerational emotional support (0.411*) influence life satisfaction which mediated intention. Additionally intergenerational instrumental support (0.347*) influence PEOU & PU which also mediated intention. emotional support also affects instrumental support significantly. |

**S3 Table Characteristics of studies investigating the impact of TEC (n=10)**

| **Author(s), year** | **Study design** | **Country** | **Study population** | **Care setting/context** | **Type of TEC** | **TEC purpose/function** | **Review focus** | **Perceived impacts** | **Measured impacts** | **Adverse impacts** | **Impact measurement methods** |
| --- | --- | --- | --- | --- | --- | --- | --- | --- | --- | --- | --- |
| Karlsen, 2018 | Qualitative | Norway | 18 OAs 60 years and above and a follow up interview with 15 and 7 family caregivers; received telecare services (less than 3 months) | Home/community telecare services; older adults and family caregivers | Telecare | Telecare for safety, alerts, reminders, independence, and ageing at home. | Adoption, sustained use, and impact | Increased safety and security by preventing and discovering accidents/fall and alerting when needed\|\| Increased independence, ageing at home through reminders |  | Privacy concerns related to camera surveillance. Technical issues such as inaccuracies in GPS tracking devices. Dependency on technology potential over-reliance on technology. Additional burden on Family caregiver responsibility | Perceived |
| Amabili, 2022 | Quantitative | Italy | 9 OAs with mean age of 82.5 years diagnosed with cognitive impairment | Home/community dementia care; older adult-caregiver dyads | Sensor and social robots | Social robot and environmental sensors to support autonomy, personal goals, and caregiver support. | Impact |  | Positive impact on achievement of personal goals Substantial improvement in ADL (at T1).  QoL there was improvement in VAS from T0 to T2 from 71(+/-19.2) to 80(+/-16.7) --- No change in average caregiver stress |  | Goal Attainment Scale (GAS). 34 ADL items VAS |
| Finch, 2017 | Quantitative | USA | 268 older adults with mean age of 79.4, 78.9 and 81.8 across three groups | Homes of older adults enrolled in a healthcare plan | Passive remote patient monitoring | Passive remote patient monitoring for health, safety, and case-manager response. | Impact |  | Less custodial care, emergency department (ED) services, inpatient stays, and ED costs than the two control groups. Mean total per-member, per-month (PMPM) costs for the intervention group were $3,649.11, compared to $4,223.43 in the declined, not-enrolled group and $4,336.24 in the control group. |  |  |
| Gordon, 2022 | Mixed method | Australia | 30 community dwelling older adults 65 and above with mean age of 79 years | Community/home context | Health monitoring, communication and entertainment devices and security alarms | Individualised technology package for health monitoring, communication, entertainment, security, and safety goals. | Acceptance/use and impact | Sense of safety and security. Increased confidence and skills with technology.  Increased communication with family and friends. | Improved well-being on personal wellbeing index (PWI): Future security from pre mean 8 to post mean 8.6 (<0.001*), Achieving in life improved from 7.3 to 7.9 (0.003*). Quality of life on Australia QoL-8D, independent living (<0.001), Self-worth (<0.001), relationships (0.001*)  COPM (Canadian Occupational Performance Measure) Goals: goal performance from 4.9 to 8,5 (<0.001*) (<0.001*) and goal satisfaction from 4.9 pre mean to 8.5 post mean (<0.001*) |  | Personal wellbeing index; QoL-8D; COPM; goal satisfaction |
| Lazarou, 2016 | Mixed method | Greece | Four OAs with cognitive impairment (2 with mild cognitive impairment and 2 dementia) | Home/community context; older adults with cognitive impairment | Assistive technology home care solutions | Assistive technology home-care solutions for cognitive and functional support. | Impact |  | Improved neuropsychological assessments across the 4 participants. The overall neurological assessment was mean=42 versus initial mean=34.75. |  | Neurological assessment |
| Igarashi, 2023 | Quantitative | Japan | 400 community dwelling older adults 65 and above: this study included 200 older adults living alone and 200 couples | Community-dwelling older adults; solo and couple-only households | Ambient sensors | Ambient sensors for household monitoring, safety, security, and daily-life support. | Acceptance/adoption and perceived impact | Older adults perceived sense of security, quality of daily life and activities has improved. |  |  |  |
| Lie, 2016 | Qualitative | UK | 24 interviews in first trial and 43 interviews in the second trial with OAs | Home monitoring trial context | SHel home monitoring system | Home monitoring system for safety, privacy-sensitive monitoring, and support for ageing in place. | Acceptability/use and impact |  |  | Concerns about privacy infringement |  |
| Lu, 2025 | Quantitative | Australia | 130 older adult participants, 61 control and 69 intervention group, mean age of 82.4 years | Home/community monitoring intervention | Home monitoring system | Home monitoring system to support social care-related quality of life. | Impact |  | At the 6-month assessment, the intervention group had a higher ASCOT score (indicating better social care-related quality of life) compared to the control group (mean difference=0.045, 95%CI=0.001-0.089). However, no difference at 12 months. And other domains were not significant including QoL |  | ASCOT |
| Pais, 2020 | Quantitative, | Switzerland | 13 older adults, 13 family care givers, 20 nurses | Home care/in-home monitoring context; older adults, caregivers, and nurses | In-home monitoring system | In-home monitoring system/sensors to support home care, safety, and quality of life. | Acceptability and impact | Help with staying at home, improving home care and quality of life, preventing domestic accidents, and reducing family stress.  Nurses reported that in-home sensors can help improve quality of life and reduce family stress. |  |  |  |
| Aggar, 2021 | Quantitative | Australia | 60 older adults between the age of 68 and 90 with mean age= 80.1 years, 48% living alone (sub-group analysis) | Community-dwelling older adults homes | SHT | Smart home technology to support quality of life, safety, and independent living. | Impact | Overall self-perceived quality of life improved (mean difference=4.02, p-value=0.01*); Achieving in life mean difference=5.6, p-value=0.026*), Future security mean difference=7, p-value=0.004*) |  |  | Perceived wellbeing index |
