# Supplementary material for: Acceptance and impact of Technology-Enabled Care (TEC) among community-dwelling older adults: A rapid review
Source: PLOS Digit Health. 2026 Aug 4;5(8):e0001625. doi: 10.1371/journal.pdig.0001625 (PMC13436759; doi:10.1371/journal.pdig.0001625)
Supplement: Supplementary file 4 [file pdig.0001625.s004.docx]

**S2 Table. Summary matrix of thematic domains and cross-cutting themes**

| Cross-cutting insight | Trust | Intrusiveness versus autonomy | Stigma and identity | Perceived control |
| --- | --- | --- | --- | --- |
| Individual readiness & response | Self-efficacy and previous experiences with TEC | Emotional tension between needing help and wanting independence. | Fear of being labelled dependent, old, or weak that discourages use. | Confidence, skills, and informed choice strengthen perceived control. |
| Perceived value & trade-offs | Concerns about data security, privacy, false alerts, and system reliability. | Monitoring feels intrusive but also enables independence | Visible technologies may alter self-image or signal decline. | When users choose TEC that aligns with their needs, it enhances autonomy. |
| Sociocultural & relational dynamics | Mediated through family, caregivers, and health providers. | Family push for surveillance out of concern, at times against user wishes | Social norms and caregiver framing influence perceived appropriateness | Negotiations within families or with staff impact users’ sense of agency |
| Contextual & structural enablers | Trust in health and social systems (training, implementation, oversight). | Systemic reliance on surveillance TEC may override user autonomy at home |  | Personalised training and customization empower users, increase ownership and satisfaction. |
| Technology design & appropriateness | Reliability and accuracy of devices; interface transparency builds or erodes trust. | Design (e.g., visible cameras, tracking) triggers feelings of being watched or controlled. | Aesthetic/design aspects (size, visibility) affect stigma; discreet designs reduce identity conflict, obtrusiveness | Customizable features, intuitive design, and flexible settings improve perceived control. |
